# Longterm Management of Portal Hypertension: Therapeutic and Prophylactic Treatment: A Panel Presentation

**DOI:** 10.1155/1991/28518

**Published:** 1991

**Authors:** John Terblanche, P. C. Bornman, K-J. Paquet, J. Mike Henderson, Yasuo Idezuki

**Affiliations:** 1 Department of Surgery University of Cape Town Medical School Observatory Cape Town 7925 South Africa; 2 Cape Town South Africa; 3 Bad Kissingen Germany; 4 Atlanta USA; 5 Tokyo Japan


*HPB Surgery*, 1991. Vol. 4, pp 1–3
Reprints available directly from the publisher
Photocopying permitted by license only

© 1991 Harwood Academic Publishers GmbH
Printed in the United Kingdom

*Symposium* **Portal Hypertension**

### LONGTERM MANAGEMENT OF PORTAL HYPERTENSION: THERAPEUTIC AND PROPHYLACTIC TREATMENT

### A PANEL PRESENTATION*

JOHN TERBLANCHE
*Cape Town, South Africa (Chairman)*

P.C. BORNMAN
*Cape Town, South Africa*

K-J. PAQUET
*Bad Kissingen, West Germany*

J. MIKE HENDERSON
*Atlanta, United States of America*

YASUO IDEZUKI
*Tokyo, Japan*

KEY WORDS: Portal hypertension, sclerotherapy, shunts, transection and devascularization, prophylactic therapy

*Presented at the 12th Annual Meeting of the International Hepato-Biliary Pancreatic Association in Hong Kong in August 1990

Correspondence to: John Terblanche, Head: Department of Surgery, University of Cape Town, Medical School, Observatory 7925, Cape Town, South Africa

### INTRODUCTION TO PORTAL HYPERTENSION SYMPOSIUM

John Terblanche, Department of Surgery and the Medical Research Council Liver Research Centre, University of Cape Town and Groote Schuur Hospital, Cape Town, South Africa.

This symposium overviews the longterm management of patients with portal hypertension. Emphasis is placed on the longterm management of patients after a variceal bleed (therapeutic management). However, the controversy whether prophylactic management should be introduced prior to the first variceal bleed, in the hope that this will reduce the incidence of bleeding and improve survival, is also addressed.

Portal hypertension management remains a major challenge and poses therapeutic dilemmas for both physicians and surgeons. This symposium includes the papers presented at the symposium at the 12th Annual Meeting of the International Hepato-Biliary-Pancreatic Association in Hong Kong in August 1990. In accepting the challenge to present a meaningful symposium both at the IHBPA Meeting and in print, it was my privilege to select a group of leading surgeons who have been involved in portal hypertension management for many years and who are from different geographic areas.

Although generally agreed that treatment is needed after a documented episode to prevent further variceal bleeding, consensus on the best management policy is lacking. It is important to emphasize that with the exception of hepatic transplantation, no other form of therapy aimed at reducing the incidence of bleeding varices treats the underlying liver disease and some (such as portosystemic shunting) may even aggravate the underlying liver disease. Oesophageal varices occur in portal hypertension caused by various aetiological conditions with very different prognosis. The prevalence of causes varies geographically and thus it is important to know the incidence of various causes in the geographic area from which results are being reported. Probably the most common cause of portal hypertension worldwide is schistosomiasis. As it is uncommon is most Western series and in the areas covered by the panelists, it is not discussed in detail. However, prognosis is usually good with proper treatment. On the other hand, alcholic cirrhosis, which is common in Western patients, has a poor prognosis irrespective of treatment, if the patient continues to abuse alcohol.

The important problem of acute variceal bleed management is not discussed as the management policy is less controversial and well defined in most institutions. A number of rare causes of portal hypertension, and the important problem of portal hypertensive gastropathy and bleeding from the gastric mucosa are also not addressed in this symposium.

The treatment options for patients with portal hypertension and oesophageal varices who have had a previous bleed include conservative medical management of the underlying liver disease awaiting a further bleed before initiating treatment; repeated injection sclerotherapy (which is the most commonly used therapy today); the construction of a portosystemic shunt; transection and devascularization operations; and specific pharmacological therapy, usually aimed at lowering portal pressure. The latter is not presented in detail in the symposium, although the views of the panelists are presented in the Discussion. Finally, the definitive treatment, which cures both the portal hypertension and the underlying liver disease, is hepatic transplantation. However, as Dr Henderson emphasises, transplantation is only applicable to a small subset of patients who have bleeding varices due to portal hypertension. Nevertheless, I believe that all patients presenting with bleeding oesophageal varices should be assessed as potential hepatic transplant recipients because the ideal pre-transplant therapy may differ from standard therapy if they are accepted onto a transplant programme. Here, sclerotherapy is the preferred treatment. If other therapy is required to salvage sclerotherapy failures the portal triad structures should be avoided, if possible, in the surgical procedures selected.

The first two authors are surgeons with extensive expertise in both sclerotherapy and major portal hypertension surgery and were asked to review sclerotherapy and the role of surgery in longterm management. Dr Henderson reviews the role of shunting and transplantation, and Dr Idezuki the role of devascularization and transection procedures. Both have made major personal contributions in these areas.

I apologise to the members of the audience who took part in that their names are not mentioned in the Discussion section because of problems with the tape recording. I take full responsibility for any deficiencies in the Discussion but gratefully acknowledge the help provided by the co-panelists in correcting and updating my version of what took place.

